# Comparison of Pronase versus Manual Dechorionation of Zebrafish Embryos for Small Molecule Treatments

**DOI:** 10.3390/jdb11020016

**Published:** 2023-03-28

**Authors:** Eva H. Hasegawa, Gist H. Farr, Lisa Maves

**Affiliations:** 1 Center for Developmental Biology and Regenerative Medicine, Seattle Children’s Research Institute, Seattle, WA 98101, USA; 2 Department of Pediatrics, University of Washington, Seattle, WA 98195, USA

**Keywords:** zebrafish, pronase, dechorionation, drug screening

## Abstract

Zebrafish are a powerful animal model for small molecule screening. Small molecule treatments of zebrafish embryos usually require that the chorion, an acellular envelope enclosing the embryo, is removed in order for chemical compounds to access the embryo from the bath medium. For large-scale studies requiring hundreds of embryos, manual dechorionation, using forceps, can be a time-consuming and limiting process. Pronase is a non-specific protease that is widely used as an enzymatic alternative for dechorionating zebrafish embryos. However, whether pronase treatments alter the effects of subsequent small molecule treatments has not been addressed. Here, we provide a detailed protocol for large-scale pronase dechorionation of zebrafish embryos. We tested whether pronase treatment can influence the efficacy of drug treatments in zebrafish embryos. We used a zebrafish model for Duchenne muscular dystrophy (DMD) to investigate whether the efficacies of trichostatin-A (TSA) or salermide + oxamflatin, small molecule inhibitors known to ameliorate the zebrafish *dmd* muscle degeneration phenotype, are significantly altered when embryos are treated with pronase versus manual dechorionation. We also tested the effects of pronase on the ability of the anthracycline cancer drug doxorubicin to induce cardiotoxicity in zebrafish embryos. When comparing pronase- versus forceps-dechorionated embryos used in these small molecule treatments, we found no appreciable effects of pronase on animal survival or on the effects of the small molecules. The significant difference that was detected was a small improvement in the ability of salermide + oxamflatin to ameliorate the *dmd* phenotype in pronase-treated embryos when compared with manual dechorionation. Our study supports the use of pronase treatment as a dechorionation method for zebrafish drug screening experiments.

## 1. Introduction

Zebrafish (*Danio rerio*) have been frequently used in small molecule treatment studies to investigate chemical toxicity and for drug discovery [1,2,3,4]. A significant advantage of using zebrafish for small molecule treatments is that the chemicals can simply be added to the bath medium to be absorbed. For treatments of zebrafish embryos, one potential barrier that may impede chemical uptake is the chorion, an acellular envelope composed of sheets of fibrillar proteins [5,6]. Although the chorion is permeable to small molecules less than 3000 Da, it restricts the uptake of larger compounds [7,8,9,10,11]. Hence, dechorionation is often a necessary step when investigating the effects of chemicals on zebrafish embryos.

Two approaches are commonly used to dechorionate zebrafish embryos: manual removal with forceps and enzymatic degradation with pronase treatment [12]. The chorion can be removed using forceps to pinch the chorion surface and pull outward to tear the chorion open and release the embryo [9,12]. Depending on the scale of the experiment, manual chorion removal can be a laborious and time-consuming task due to the care and delicacy required. Another common approach is to use pronase, a mixture of proteolytic enzymes isolated from *Streptomyces griseus*, to enzymatically soften the chorion to the point where it ruptures, releasing the embryo [12,13,14]. This process involves incubating the embryos in a solution with pronase while periodically agitating it to dislodge the embryos from the chorions [9,10,11,12,13,14,15,16]. Because many embryos can be treated simultaneously, pronase treatments can be more time-efficient compared with manual dechorionation.

Despite pronase treatment being a commonly used method for zebrafish embryo dechorionation, there is limited standardization of protocols with respect to the concentration and duration of pronase exposure. One concern is that prolonged exposure to pronase may cause damage to the embryos themselves and result in high mortality [9,14,17,18]. Additionally, pronase exposure may influence how embryos subsequently respond to chemical treatments [14,18]. To address these concerns, the aims of this study were to (1) provide a detailed protocol for dechorionating zebrafish embryos with pronase for large-scale small molecule treatments, and (2) compare pronase- versus forceps-dechorionated embryos exposed to small molecule treatments. We used treatments of the HDAC inhibitor trichostatin A (TSA) or the combination of the epigenetic inhibitors salermide + oxamflatin for our comparison of dechorionation procedures because of the well-documented ability of these small molecules to ameliorate muscle lesions in an established zebrafish model for Duchenne muscular dystrophy (DMD) [19,20,21]. We also used treatments of doxorubicin, a cancer drug with cardiotoxic effects, for comparison of the dechorionation procedures because of its well-documented ability to induce cardiotoxicity in zebrafish embryos [22,23,24,25].

## 2. Materials and Methods

### 2.1. Solutions

Recipes for the solutions used in this study are provided as follows:
**ICS Water****Reagent****Quantity****Final Concentration**Instant Ocean Salt5.97 g300 mg/LCaCl_2_1.26 g0.56 mMNaHCO_3_1.9 g1.2 mMRO waterUp to 20 L



**Pronase Stock Solution**

**Reagent**

**Source**

**Quantity**

**Final Concentration**
Pronase from *Streptomyces griseus*Roche Applied Science1 g20 mg/mLRO water
Up to 50 mL
Make 1 mL aliquots and store at −20 °C.


**Embryo Medium 20X Stock Solution**

**Reagent**

**Quantity**

**Final Concentration**
NaCl17.5 g15 mMKCl0.75 g0.50 mMCaCl_2_·2H_2_O2.9 g1 mMKH_2_PO_4_0.41 g0.15 mMNa_2_HPO_4_0.142 g0.05 mMMgSO_4_·7H_2_O4.9 g1 mMRO waterUp to 1 L
Vacuum filter and store at 4 °C.


**Sodium Bicarbonate 1000X Stock Solution**

**Reagent**

**Quantity**

**Final Concentration**
NaHCO_3_0.70 g0.83 MRO waterUp to 10 mL
Make 1 mL aliquots and store at −20 °C.


**Embryo Medium 1X Stock Solution**

**Reagent**

**Quantity**

**Final Concentration**
20X Embryo medium50 mL1X1000X Sodium bicarbonate1 mL0.83 mMRO waterUp to 1 L
Store at room temperature and use within 1 week.


**TSA Treatment Solution**

**Reagent**

**Source**

**Quantity**

**Final Concentration**
TSA, 1 mM in DMSOSigma-Aldrich12 μL200 nMDMSOSigma-Aldrich48 μL0.0704 M (0.5%)1X Embryo medium
Up to 12 mL
Make fresh each day.


**Salermide + Oxamflatin Treatment Solution**

**Reagent**

**Source**

**Quantity**

**Final Concentration**
Salermide + oxamflatin, 1 mM each in DMSOCayman Chemical60 μL5 μM each1X Embryo medium
12 mL
Make fresh each day.


**Doxorubicin treatment solution**

**Reagent**

**Source**

**Quantity**

**Final concentration**
Doxorubicin, 10 mM in DMSOSigma-Aldrich10 μL100 μM1X Embryo medium
1 mL
Make fresh each day.


**DMSO Control Treatment Solution**

**Reagent**

**Source**

**Quantity**

**Final Concentration**
DMSOSigma-Aldrich60 μL0.0704 M (0.5%)1X Embryo medium
Up to 12 mL
Make fresh each day.

### 2.2. Zebrafish Husbandry

All of the experiments involving live zebrafish (*Danio rerio*) were carried out in compliance with Seattle Children’s Research Institute’s Institutional Animal Care and Use Committee guidelines. Zebrafish were raised and staged as previously described [12,21]. Time (hpf or dpf) refers to hours or days post-fertilization at 28.5 °C. The eggs were collected from 20–30 min spawning periods and raised in Petri dishes in ICS water in a dark 28.5 °C incubator, up to 5 dpf. After 5 dpf, the fish were maintained on a recirculating water system (Aquaneering) under a 14 h on, 10 h off light cycle. From 6–30 dpf, the fish were raised in 2.8 L tanks with a density of no more than 50 fish per tank and were fed a standard diet of paramecia (Carolina) one time per day and Zeigler AP100 dry larval diet two times per day. From 30 dpf onwards, the fish were raised in 6 L tanks with a density of no more than 50 fish per tank and were fed a standard diet of Artemia nauplii (Brine Shrimp Direct) and Zeigler adult zebrafish feed, each two times per day. The wild-type stock and genetic background used was AB. The zebrafish *dmd^ta222a^* mutant line (also known as *sapje*; hereafter referred to as *dmd*) has been described and is a null allele [26,27]. *dmd^ta222a^* genotyping was performed as previously described [28]. The *Tg(myl7:EGFP)^twu34^* line has been previously described [29].

### 2.3. Dechorionation

Zebrafish that were heterozygous for the *dmd*^ta222a^ mutation were crossed as groups, as previously described [12]. The eggs were thoroughly rinsed with ICS water, sorted to remove dead or unfertilized eggs, placed in 10 cm plastic Petri dishes with a maximum of 100 embryos each, and raised at 28.5 °C. Dechorionation was performed at 24 hpf immediately before starting the drug treatments.

**Figure 1:**
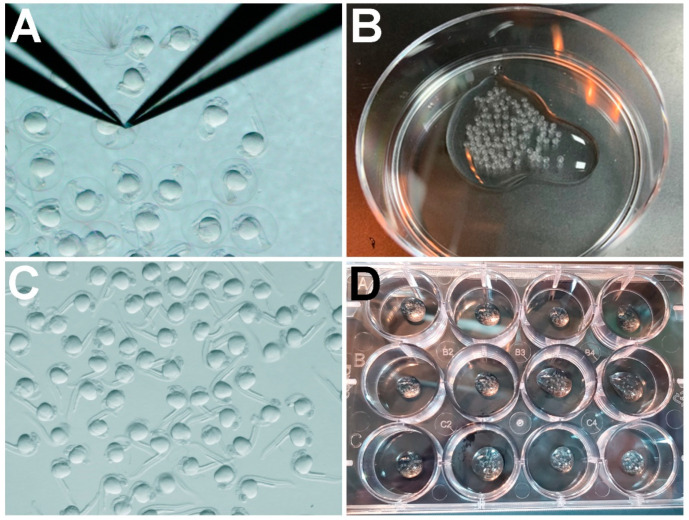
Zebrafish embryo dechorionation steps. (**A**) Using forceps to remove the chorions of 24 hpf embryos: (**B**) 24 hpf embryos transferred into a 60 mm × 15 mm dish prior to pronase treatment; (**C**) 24 hpf embryos immediately after 14 min treatment with 0.5 mg/mL pronase and 3X rinse with ICS water; (**D**) dechorionated 24 hpf embryos in a 12-well plate prior to treatment with TSA or control DMSO.

To manually dechorionate, we used Dumont #5 forceps [12]. The forceps were used to pinch the chorion and pull outwards, tearing open the chorion to release the embryo, while making sure not to damage the embryo (Figure 1A).

Pronase dechorionation was performed as follows:

1. To make 0.5 mg/mL pronase solution, mix 125 µL of 20 mg/mL pronase stock solution in 5 mL RO water in a 15 mL tube.
2. Using a Pasteur pipette, transfer up to 100 24 hpf embryos to a 60 mm × 15 mm Petri dish (Figure 1B). Remove excess ICS water.
3. Pour in 5 mL of 0.5 mg/mL pronase solution per dish and immediately start a timer set for 14 min.
4. Swirl the dish 3−4X during the treatment incubation. A few chorions may begin coming off during the last 2–3 min of incubation.
5. Immediately after the timer goes off, carefully pour off most of the liquid into a waste container. Be sure to not lose any embryos. Some liquid can be left in the dish.
6. Rinse the embryos by immediately adding about 10 mL of ICS water to the dish. A plastic wash bottle may be used.
7. Using a plastic transfer pipette (Fisher Scientific 13-711-7M), very gently pipette the ICS water and embryos up and down about 5X to remove any remaining chorions.
8. Repeat the ICS water rinse two more times to remove chorions and residual pronase.
9. Using a stereomicroscope, confirm that all of the embryos have been released from their chorions and are undamaged (Figure 1C).

### 2.4. Drug Treatments

TSA, salermide + oxamflatin, and DMSO (control) treatment solutions were prepared fresh on the day of treatments. For TSA and salermide + oxamflatin treatments, the embryos from crosses of heterozygous *dmd* fish were used. At 24 hpf, 25 dechorionated embryos per well were transferred into empty wells of a 12-well plate, using a Pasteur pipette and waiting for the embryos to settle at the bottom of the pipette in order to transfer them in a minimal volume (Figure 1D). The excess medium was removed using a Pasteur pipette and 3 mL of drug or 3 mL of DMSO treatment solution was added to the wells. Plates were then placed in a 28.5 °C incubator. The treatment solutions were replaced daily at about 48 hpf and 72 hpf by preparing fresh treatment solutions, removing most (approximately 2.8 mL) of the old treatment solution, and adding 3 mL fresh solution. At about 96 hpf, the embryos were fixed in 4% PFA in 1X PBS for subsequent genotyping and measurement of the birefringence. For the doxorubicin treatments, embryos from crosses of *Tg(myl7:EGFP)* fish were used. Eight embryos per well of 24-well plates were treated in a volume of 1 mL. Treatments started at 24 hpf and the solutions were changed at about 48 hpf and 72 hpf. The embryos were fixed at 96 hpf for phenotypic scoring.

### 2.5. Imaging and Quantitation of Muscle Lesions

Imaging of larval muscle using polarized light and quantitation of muscle birefringence were performed as previously described [21]. To briefly summarize the approach, after fixation, the larvae were rinsed in PBS with 0.01% Tween (PBSTw) and placed in 2.5% methyl cellulose in a 60-mm glass Petri dish for imaging. An Olympus SZX16 stereomicroscope with an attached Olympus DP72 camera was set up with one sheet of polarizing film over the trans-illumination base and another sheet of the film placed over the objective lens, such that the two films were crossed. The larvae were oriented to maximize the brightness of the muscle tissue through the crossed polarizers. Images were acquired using Olympus Cellsens Dimensions software. ImageJ was used to outline the trunk musculature and to calculate the average pixel intensity within the resulting selection. The average pixel intensity values for experimental conditions were normalized to the wild-type control values.

## 3. Results

### 3.1. Determination of Pronase Treatment Specifications

We used an existing protocol [16] to provide the basis for testing the variables of the pronase treatment. For each treatment test, 5 mL of pronase treatment solution was used on 100 24 hpf embryos in 60 mm Petri dishes. We focused on 24 hpf embryos because that is a typical stage for initiating small molecule treatments and screens [21,23,30].

We first tested 1 mg/mL pronase treatments with treatment durations of 2.5 min, 4 min, and 6 min. When exposed for 2.5 min, almost no chorions were removed. After 6 min, the chorions could be removed only after repeated and vigorous pipetting of the embryos.

We then tested 0.5 mg/mL pronase treatments with treatment durations of 10 min, 12 min, and 14 min. While the 10 and 12 min exposure times with 0.5 mg/mL improved dechorionation, repeated pipetting was still required. However, the 14 min exposure resulted in essentially 100% of the embryos losing their chorions with only very minimal pipetting (see Materials and Methods). Additionally, almost no mortality was observed due to the pronase treatments, even after following the embryos for four days after pronase treatment (average 99.6% survival, *n* = 5 dishes of 100 embryos each). We conclude that treating with 0.5 mg/mL pronase solution for 14 min is optimal for dechorionation of batches of 100 24 hpf embryos.

### 3.2. Small Molecule Treatments of dmd Embryos Exhibit Similar Rescue Following Either Manual or Pronase Dechorionation

**Figure 2:**
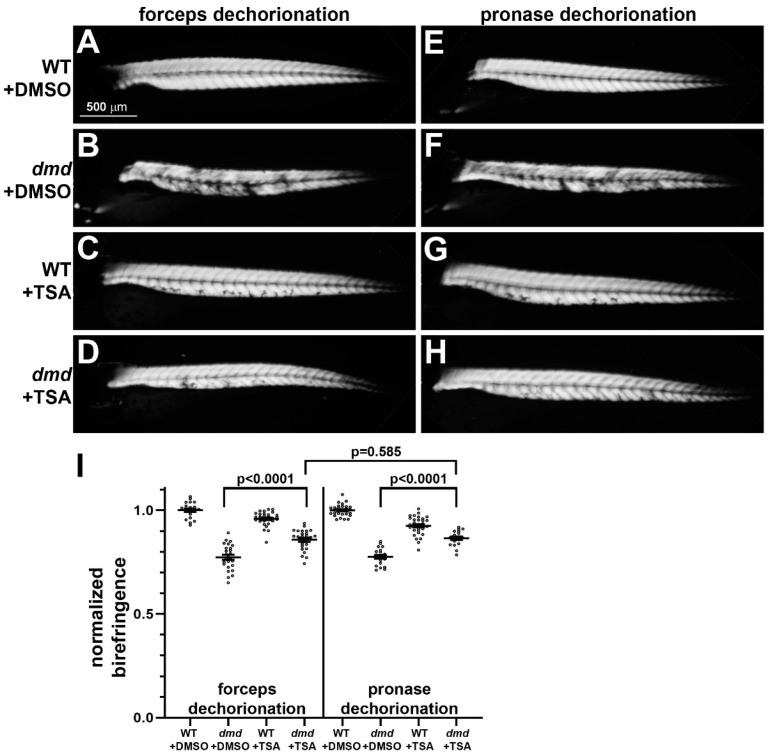
Muscle birefringence of TSA-treated zebrafish larvae following manual or pronase dechorionation. (**A**–**H**) Zebrafish trunk skeletal muscle birefringence at 4 dpf. Lateral views, anterior to the left. Images show (**A**,**E**) *dmd*+/+ wild-type (WT) + DMSO, (**B**,**F**) *dmd*+/+ WT + TSA, (**C**,**G**) *dmd*−/− (*dmd*) + DMSO, and (**D**,**H**) *dmd*−/− (*dmd*) + TSA. (**I**) Graph of the average normalized birefringence pixel intensities (mean grey values) in WT (*dmd*+/+) and *dmd* (*dmd*−/−) zebrafish larvae treated with DMSO (control), and dechorionated with forceps (left) or pronase (right). Horizontal lines represent the mean and standard error of the mean. Each dot represents an individual embryo (*n* = 18–31 animals per condition). Mean gray values between *dmd* + DMSO and *dmd* + TSA animals were significantly different for each dechorionation condition. The mean grey values between *dmd* + TSA animals were not significantly different when comparing each dechorionation condition. Error bars represent the standard error. Significance was determined using a one-way ANOVA test comparing each treatment group to the *dmd* DMSO control group with Dunnett’s correction for multiple comparisons. Data in support of (**I**) is available in Table S1 in Supplementary Materials.

We next tested whether pronase dechorionation influences the ability of TSA treatment to ameliorate the muscle lesion phenotype in zebrafish *dmd* embryos. TSA treatments were performed as previously described [19,21], with a dose of 200 nM used from 24 hpf to 4 dpf. Immediately prior to initiating the TSA or DMSO control treatments, embryos were either manually dechorionated with forceps or pronase treated with 0.5 mg/mL for 14 min. Similar to what we have shown previously [19,21], following manual dechorionation, homozygous mutant *dmd* embryos treated with TSA showed significantly improved muscle structure compared with DMSO control-treated *dmd* embryos (Figure 2A–D,I). Following pronase dechorionation, *dmd* embryos treated with TSA also showed improved muscle structure (Figure 2E–I). The ability of TSA treatment to improve the *dmd* muscle lesion phenotype, as measured by quantitative muscle birefringence [21], showed no significant difference when comparing forceps- versus pronase-dechorionated embryos (Figure 2I). Among the animals that were dechorionated with forceps, 100% survived in both DMSO and TSA conditions (*n* = 100 per condition). Among the animals that underwent pronase treatment, 99% survived in the DMSO condition and 93% survived in the TSA treatment (*n* = 100 per condition). Thus, pronase treatment did not appear to substantially influence the effects of TSA treatment when compared with manually dechorionated embryos.

**Figure 3:**
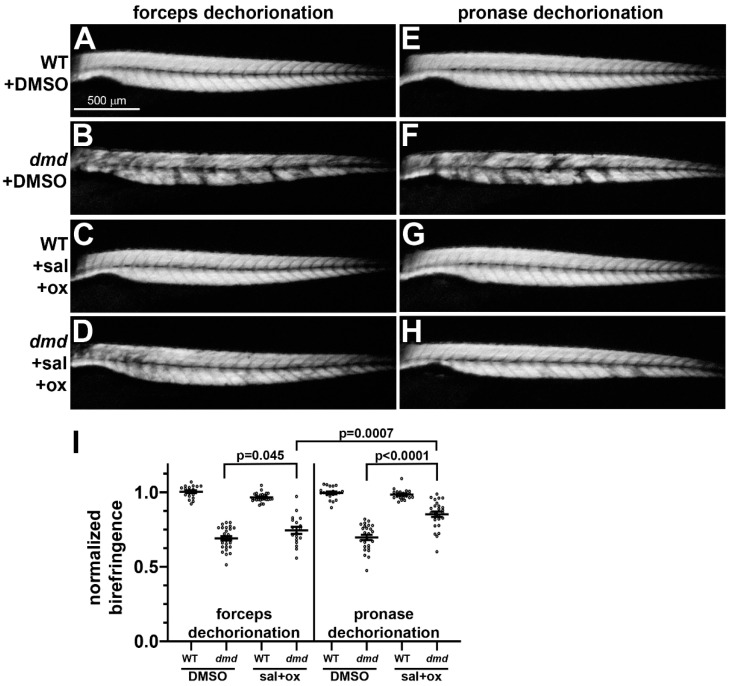
Muscle birefringence of salermide + oxamflatin-treated zebrafish larvae following manual or pronase dechorionation. (**A**–**H**) Zebrafish trunk skeletal muscle birefringence at 4 dpf. Lateral views, anterior to the left. Images show (**A**,**E**) *dmd*+/+ wild-type (WT) + DMSO, (**B**,**F**) *dmd*+/+ WT + salermide+oxamflatin, (**C**,**G**) *dmd*−/− (*dmd*) + DMSO, and (**D**,**H**) *dmd*−/− (*dmd*) + salermide+oxamflatin. (**I**) Graph of average normalized birefringence pixel intensities (mean grey values) in WT (*dmd*+/+) and *dmd* (*dmd*−/−) zebrafish larvae treated with DMSO (control) or salermide + oxamflatin and dechorionated with forceps (left) or pronase (right). Horizontal lines represent the mean and standard error of the mean. Each dot represents an individual embryo (*n* = 18–28 animals per condition). Mean gray values between *dmd* DMSO and *dmd* salermide + oxamflatin animals were significantly different for each dechorionation condition. Mean grey values for *dmd* salermide + oxamflatin pronase-dechorionated animals were significantly higher than those for *dmd* salermide + oxamflatin forceps-dechorionated animals. Error bars represent the standard error. Significance was determined using a one-way ANOVA test comparing each treatment group to the *dmd* DMSO control group with Dunnett’s correction for multiple comparisons. Data in support of (**I**) is available in Table S2 in Supplementary Materials.

A salermide + oxamflatin mixture was used as an additional small molecule treatment to determine whether dechorionation methods influenced rescue of the *dmd* phenotype. Salermide + oxamflatin treatments were performed as previously described [21], with a dose of 5 µM used from 24 hpf to 4 dpf. Immediately prior to initiating the treatments, embryos were either manually dechorionated with forceps or pronase treated with 0.5 mg/mL for 14 min. Similar to what we have shown previously [21], following manual dechorionation, homozygous mutant *dmd* embryos treated with salermide + oxamflatin showed a significantly improved muscle structure compared with DMSO control-treated *dmd* embryos (Figure 3A–D,I). Following pronase dechorionation, *dmd* embryos treated with salermide + oxamflatin also showed improved muscle structure, with even slightly more improvement seen than after manual dechorionation (Figure 3E–I). For animals that were treated with DMSO, we observed 100% survival with both forceps and pronase dechorionation (*n* = 100 per condition). Among the animals that were treated with salermide + oxamflatin, we observed 99% survival with both forceps and pronase dechorionation (*n* = 100 per condition). Thus, pronase treatment did not inhibit the effects of salermide + oxamflatin and may have enhanced their ability to improve *dmd* muscle structure, when compared with manual dechorionation.

### 3.3. Doxorubicin Induces Cardiotoxicity Following Either Manual or Pronase Dechorionation

**Figure 4:**
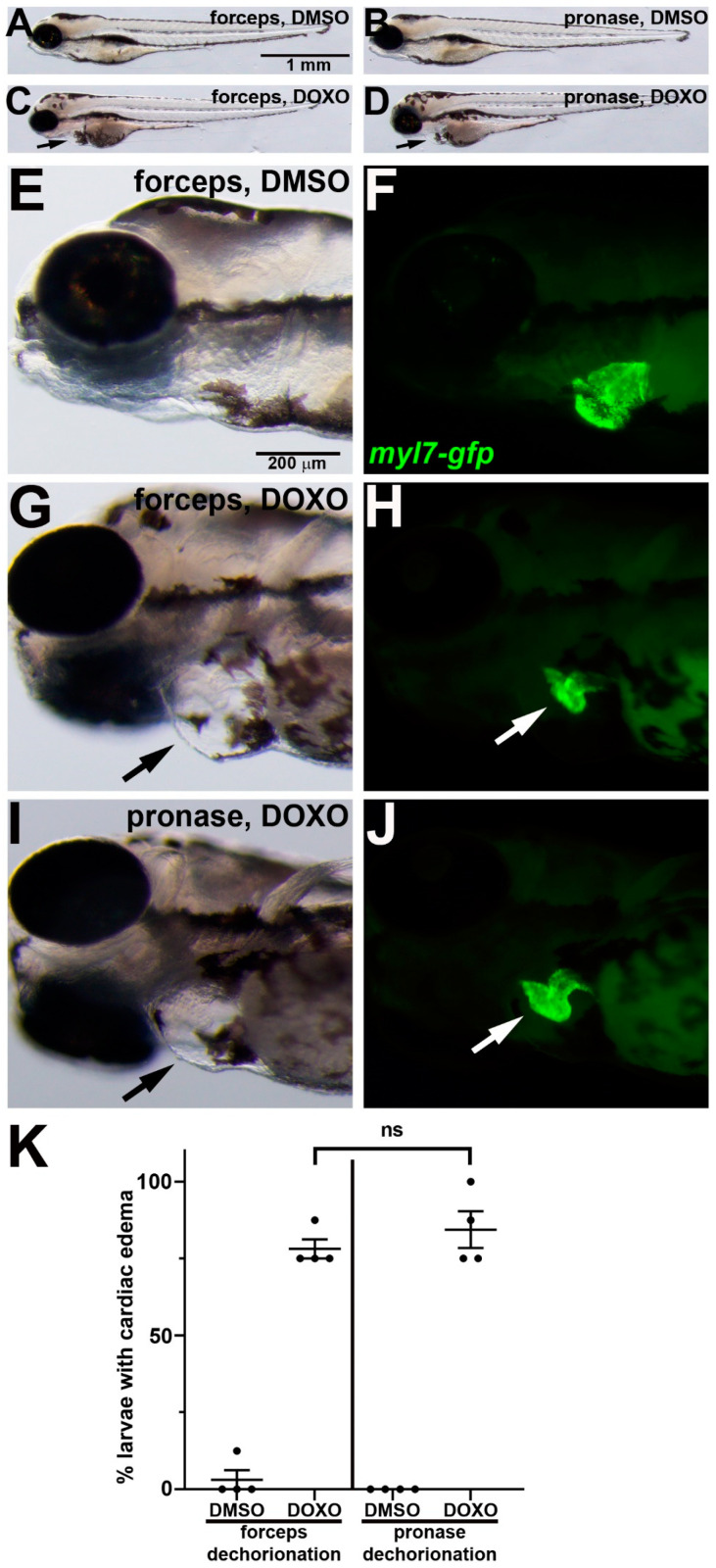
DOXO-induced cardiotoxicity in zebrafish embryos following manual or pronase dechorionation. (**A**–**J**) 96 hpf larvae that were treated from 24 hpf with (**A**,**B**,**E**,**F**) DMSO or (**C**,**D**,**G**–**J**) DOXO. Lateral views of larvae show anterior to the left. (**E**–**J**) Magnifications of head and cardiac region show paired brightfield and fluorescent images. Black arrows in (**C**,**D**,**G**,**J**) point to swelling around the heart. In (**F**,**H**,**J**), *myl7-gfp* (green) labels the myocardium, which is reduced in DOXO treatment ((**H**,**J**), white arrows). (**K**) Graph of % larvae showing cardiac edema. *n* = 4 treatment replicates, each consisting of eight embryos. ns = not significant.

We next tested whether pronase dechorionation influences the ability of doxorubicin (DOXO) treatment to induce cardiotoxicity in zebrafish embryos. DOXO treatments were performed as previously described [23,25], with a dose of 100 µM used from 24 hpf to 3 dpf. Immediately prior to initiating the DOXO or DMSO control treatments, embryos were either manually dechorionated with forceps or pronase treated with 0.5 mg/mL for 14 min. Similar to what has been shown previously [23,25], following manual dechorionation, DOXO-treated embryos showed swelling around the heart cavity and reduced myocardial tissue compared with DMSO control-treated embryos (Figure 4A,C,E–H). Following pronase dechorionation, DOXO-treated embryos showed the same cardiac defects (Figure 4B,D,I–K). Among the animals that underwent forceps dechorionation or pronase dechorionation, 100% survived in both DMSO and DOXO conditions (*n* = 32 animals per condition). Thus, pronase treatment did not appear to influence the effects of doxorubicin treatment when compared to manually dechorionated embryos.

## 4. Discussion

We undertook this study as part of our efforts to optimize our protocol for small molecule treatments of zebrafish embryos. We sought to improve the throughput of the dechorionation step by comparing enzymatic dechorionation using pronase to our standard method of manual dechorionation using forceps. Pronase is an accepted enzymatic approach used to remove zebrafish embryo chorions [12,14]. However, there is limited standardization of protocols with respect to the concentration and duration of pronase exposure. We thus wanted to optimize pronase treatment conditions to achieve complete dechorionation with minimal handling and minimal damage to the embryos for use in large-scale drug treatment experiments.

Pronase concentrations previously reported for use in zebrafish embryos include 0.1 mg/mL for 6.5 min (4 hpf embryos) [14], 1 mg/mL for 5–10 min (17 hpf embryos) [16], and 2 mg/mL for 1 min [12]. In addition to these different concentrations and treatment durations, most protocols describe employing additional agitation steps, although these are not always well defined [9,12,14,16]. Our protocol, optimized for concentration and treatment duration, uses minimal agitation to dislodge the embryos, thus enhancing the efficiency of the dechorionation step without damaging the embryos or affecting their subsequent development.

One concern with using enzymatic dechorionation is that exposure to pronase may cause damage to the embryos themselves and result in high mortality [9,14,18,31]. Pronase treatments of 1 mg/mL on 6 hpf zebrafish embryos have been shown to cause high mortality [9]. Similarly, Nile tilapia embryos treated with pronase at the cleavage and blastula stages did not survive to the hatching stage [17]. However, treatment of 24 hpf zebrafish embryos with 2 mg/mL pronase for 10 min did not significantly alter heart rate, a metric of developmental stress [32]. With our protocol, 24 hpf embryos were successfully dechorionated with 0.5 mg/mL pronase, without any apparent damage, developmental delay, or effect on survival to 4 dpf. Whether younger stage zebrafish embryos can be safely dechorionated with our pronase protocol remains to be tested.

Homozygous mutant *dmd* embryos treated with TSA or salermide + oxamflatin showed substantial rescue of the muscle structure phenotype, regardless of the dechorionation method (Figure 2 and Figure 3). These results support our previous findings that TSA and salermide + oxamflatin ameliorate *dmd* muscle lesion severity [19,21]. We did observe two possible deviations in these experiments in the effects of pronase compared with forceps dechorionation. Embryos that underwent pronase dechorionation and TSA treatment did show a somewhat lower survival compared with manually dechorionated embryos treated with TSA (Figure 2). In addition, embryos that underwent pronase dechorionation and salermide + oxamflatin treatment showed significantly greater restoration of muscle fiber integrity compared with manually dechorionated embryos treated with salermide + oxamflatin (Figure 3). These differences may reflect some variability between TSA or salermide + oxamflatin treatments or may indicate that pronase treatment facilitates the effects of salermide + oxamflatin through an unknown mechanism. To further test for the influence of pronase on other small molecule treatments, we asked whether pronase treatments altered the cardiotoxic effects of doxorubicin. We found no detectable difference in the effects of doxorubicin treatments following manual or pronase dechorionation (Figure 4). Our experiments show that pronase treatment did not appear to strongly inhibit or otherwise alter the effects of these different small molecules in zebrafish. Whether pronase treatment influences the activity of other small molecule treatments of zebrafish embryos remains to be tested. For future studies, we recommend that researchers consider whether enzymatic dechorionation might influence the effects of the particular drug treatments or downstream assays being performed on zebrafish embryos.

In conclusion, incubating batches of 100 24 hpf embryos in 0.5 mg/mL pronase for 14 min is an effective dechorionation method. Pronase treatment did not strongly alter the effects of three different small molecule treatments when compared with manually dechorionated embryos. To the best of our knowledge, this is the first study to directly compare pronase versus manual dechorionation for zebrafish embryo small molecule treatments. Our study supports the use of pronase treatment as an effective and efficient method for large-scale dechorionation of 24 hpf zebrafish embryos for small molecule screening experiments.

## Data Availability

The data presented in this study are contained within the article or are available in Supplementary Data Tables.

## Supplementary Materials

The following supporting information can be downloaded at: https://www.mdpi.com/article/10.3390/jdb11020016/s1, Table S1. Data supporting Figure 2I, Table S2. Data supporting Figure 3I.
